# Secondary Immune Thrombocytopenic Purpura Triggered by COVID-19

**DOI:** 10.7759/cureus.14505

**Published:** 2021-04-15

**Authors:** Abi Watts, Kavin Raj, Pooja Gogia, Christian C Toquica Gahona, Marcus Porcelli

**Affiliations:** 1 Internal Medicine, Saint Peter’s University Hospital, New Brunswick, USA; 2 Hematology and Oncology, Saint Peter’s University Hospital, New Brunswick, USA

**Keywords:** immune thrombocytopenic purpura, covid 19, thrombocytopenia

## Abstract

Multiple infectious causes have been implicated with the development of secondary immune thrombocytopenic purpura (ITP). Nevertheless, new pathogens, including coronavirus disease 2019 (COVID-19), are recently being described in its development. A 41-year-old Hispanic male presented to the Emergency Department with a two-day history of bleeding gums and blood-tinged sputum. A severe acute respiratory syndrome coronavirus 2 (SARS-CoV-2) polymerase chain reaction (PCR) test was positive on admission. Initial laboratory studies showed severe thrombocytopenia of 3x10^9^/L (150-400x10^9^/L) with no abnormal platelets or schistocytes seen on peripheral blood smear, with normal prothrombin time/international normalized ratio (PT/INR), partial thromboplastin time (PTT) and fibrinogen levels. Secondary causes of thrombocytopenia were ruled out. One unit of single donor platelets was transfused and the patient was treated with intravenous dexamethasone for a total of five days and intravenous immunoglobulin (IVIG) for two days. One week after discharge the patient had a recurrence of epistaxis and hematuria requiring a second course of steroids and IVIG and the decision was made to start the patient on eltrombopag 50mg daily, which maintained his platelet counts within normal limits. COVID-19-associated ITP can be severe and life-threatening and hence warrants rapid and prompt management with steroids and IVIG. In refractory cases, thrombopoietin receptor agonists should be used.

## Introduction

Immune thrombocytopenia purpura (ITP) is a rare disease characterized by isolated low platelet counts (less than 100 x10^9^/L) in absence of secondary causes that lead to an increased risk of bleeding. It is well known that many viruses like cytomegalovirus, varicella zoster virus, HIV and hepatitis C virus cause secondary ITP [1]. Nonetheless, the relationship between coronavirus disease 2019 (COVID-19) as an inciting factor of ITP is still not well recognized. We present a patient affected by COVID-19-induced ITP.

## Case presentation

A 41-year-old male presented to the Emergency Department (ED) with a two-day history of bleeding gums and blood-tinged sputum. The patient had a one-week history of fever, cough, body aches, and difficulty breathing. There was no previous history of gum bleeding, hemoptysis, epistaxis, spontaneous joint swelling, or prolonged bleeding after an injury. He had no family history of blood disorders and he was not taking any medications including antiplatelet or anticoagulant drugs. He denied the use of tobacco, alcohol, recreational drugs, or herbal supplements.

At presentation, he was febrile with a temperature of 102.8 F, respiration rate of 32 breaths/min, heart rate 92 beats/min, and oxygen saturation 88-89% on room air. Physical exam was notable for maroon-colored exudates in his mouth indicative of gum bleeding and decreased bilateral breath sounds with bibasilar crackles, with no scleral icterus, petechiae, ecchymoses, joint swelling, or hepato-splenomegaly. A complete blood count revealed severe thrombocytopenia of 3x10^9^/L (150-400x10^9^/L) and no abnormal platelets or schistocytes were seen on peripheral blood smear. Other labs included normal levels of prothrombin time/international normalized ratio (PT/INR), partial thromboplastin time (PTT), and fibrinogen. A chest X-ray was consistent with multifocal pneumonia. The patient tested positive for severe acute respiratory syndrome coronavirus 2 (SARS-CoV-2) by reverse transcription polymerase chain reaction (RT-PCR). His inflammatory markers (c-reactive protein [CRP], lactate dehydrogenase [LDH], and D-dimer) were within normal limits. Other secondary causes of thrombocytopenia were ruled out including HIV, hepatitis B and C, *H. pylori*, thyroid function tests, and generalized autoimmune testing with antinuclear antibodies (ANA) titer screen (Table 1). A review of previous hospital records identified that the patient had platelet counts of 122 and 54x10^9^/L (150-400x10^9^/L) respectively on two different occasions before the current visit, but no history of previous bleeding disorders.

**Table 1:** Laboratory investigations on admission SARS-CoV-2: severe acute respiratory syndrome coronavirus 2, PCR: polymerase chain reaction, TSH: thyroid stimulating hormone, T4: thyroxine, ANA: antinuclear antibodies

Test	Results
Total leucocyte count (normal: 4.5 to 11 X 10^9^/liter)	5.4
Absolute lymphocyte count (normal: 0.9 -2.90 X 10^9^/liter)	1.13
Red blood cell count (normal:4.2 to 5.4 million per microliter)	4.87
Hemoglobin (normal: 13.5 to 17.5 grams per deciliter)	13.2
Total platelet count (normal: 150 to 400 X 10^9^/ liter	3
Mean platelet volume (normal: 7.5 to 12 femtoliter)	10.3
Prothrombin time (normal: 11 to 13.5 seconds)	13.1
International normalized ratio	1.13
Partial Thromboplastin Time (normal: 28.4-37.9 seconds)	31.9
Fibrinogen (normal: 200 to 400 milligrams per deciliter)	256
D-dimer (0 to 200 nanogram per milliliter)	<150
C- reactive protein (<10 milligrams per liter)	14
Lactic acid dehydrogenase (normal: 140 to 280 units per liter)	207
SARS-CoV-2 PCR	Positive
HIV 4^th^ generation combined test (p24 and HIV-1/2 antibody)	Negative
TSH (0.465-4.68 Uiu/ml)	0.965
T4 (0.64-1.79ng/dl)	1.24
Vitamin B12 (180-914 pg/ml)	577
Folate (>2.8ng/ml)	15.8
Ferritin (18-464ng/ml)	344.3
Hepatitis C antibody	Nonreactive
ANA Screen	Negative
Streptococcus pneumonia urine antigen	Negative
Legionella urine antigen	Negative
H. pylori stool antigen	Negative
Influenza rapid antigen A and B	Negative

The patient received one unit of single donor platelet in the ED which failed to increase his platelet count. Secondary ITP triggered by COVID-19 was suspected for which hematology was consulted and recommended treatment with pulse dose of dexamethasone 40mg intravenous daily for a total of five days and intravenous immunoglobulin (IVIG) 1g/Kg for two days. He also received hydroxychloroquine plus azithromycin for COVID-19 pneumonia following the hospital protocols at the beginning of the pandemic. His platelet counts improved, and there were no further episodes of mucosal bleeding by day three of hospitalization; eventually he was discharged home.

**Figure 1:**
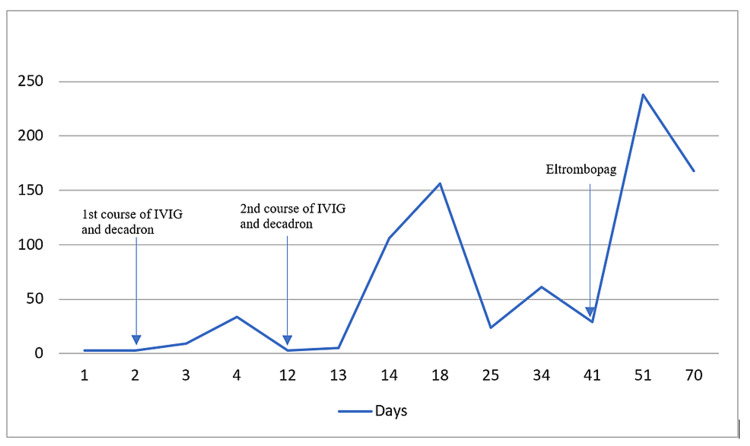
Course of platelet count Platelet counts in 109/L on Y-axis and number of days after diagnosis on X-axis. IVIG: intravenous immunoglobulin

One week after discharge, the patient developed epistaxis, hematuria, and diffuse petechiae and was readmitted to the hospital. Laboratory investigations showed isolated thrombocytopenia of 3x10^9^/L (150-400x10^9^/L) and large blood with packed red blood cells in the urine. He was restarted on a five-day course of dexamethasone and one dose of IVIG. His platelet count improved, and his bleeding resolved. The patient was discharged home on a prolonged steroid taper, and weekly complete blood count draws. His platelet count remained stable for two weeks but decreased again, after which a decision was made to start treatment with eltrombopag 50mg daily. This improved his platelet counts to normal limits with maintenance after one month of follow-up (Figure 1).

## Discussion

Several mechanisms have been proposed for thrombocytopenia in patients with COVID-19. These include direct invasion of bone marrow cells, platelet-virus interaction via pathogen recognition receptors (PRR), sepsis, secondary hemophagocytosis caused by cytokine storm, autoantibodies and immune complexes against platelets, reduced platelet release from megakaryocytes in pulmonary circulation due to damaged lung tissue and microthrombi formation and consumptive coagulopathy due to increased platelet activation in damaged lung tissue [2,3].

The most important mechanism for the development of ITP by viruses is molecular mimicry between viral components and platelet glycoproteins [4]. In a study by Zhang et al., it was demonstrated that several hepatitis C core-envelope peptides shared sequences with glycoprotein IIIa found on platelets. This molecular mimicry induces the production of antibodies which acquire the ability for platelet fragmentation [5]. As of now, no sequence homology between SARS-CoV-2 and platelet components has been described, and the underlying pathogenesis remains obscure.

Dexamethasone and IVIG are considered first-line therapies for the treatment of ITP associated with bleeding symptoms. Dexamethasone increases apoptosis of autoantibody-producing lymphocytes and causes downregulation of macrophage activity responsible for platelet phagocytosis. IVIG blocks the phagocytic destruction of platelets in the spleen [1]. Intravenous anti-D (50-75 mg/kg once) can be used as an alternative to IVIG in Rh-positive patients. If anti-D is used, consideration must be given to potential complications of disseminated intravascular coagulation (DIC) or hemolysis [6]. Other treatment options include rituximab and thrombopoietin receptor agonists (eltrombopag, avatrombopag, romiplostim) for patients refractory to steroids and IVIG. Eltrombopag is a platelet growth factor that may be used to boost platelet production. The use of platelet growth factors should be carefully evaluated, taking into account the potential thrombotic events during coronavirus infection.

Secondary ITP is associated with certain viral infections. We describe a case of COVID-19-induced ITP exacerbation. Our patient probably had primary ITP in the past given his prior low platelet counts, but never had any bleeding episodes. Infection with COVID-19 was probably the inciting event that triggered the episode of symptomatic and refractory ITP in this case. Further studies are required to support this relationship.

## Conclusions

Infection with COVID-19 may predispose patients to develop thrombocytopenia through a wide variety of mechanisms. Treatment with steroids and IVIG is required in patients with suspected COVID-19-associated ITP which can lead to severe and life-threatening hemorrhagic complications. Thrombopoietin receptor agonists should be used for patients refractory to standard therapies. 
